# The Impact of Task Context on Pleasantness and Softness Estimations: A Study Based on Three Touch Strategies

**DOI:** 10.3390/bs15010063

**Published:** 2025-01-13

**Authors:** Binyue Gao, Yinghua Yu, Yoshimichi Ejima, Jinglong Wu, Jiajia Yang

**Affiliations:** Graduate School of Interdisciplinary Science and Engineering in Health Systems, Okayama University, 3-1-1 Tsushima-Naka, Kita-ku, Okayama 700-8530, Japan; p8t27rjy@s.okayama-u.ac.jp (B.G.);

**Keywords:** pleasantness, softness, touch strategy, task context, psychophysics

## Abstract

This study investigated the two distinct perceptions (pleasantness and softness) of deformable stimuli with different degrees of compliance under conditions with and without a contextual task. Three tactile strategies—grasping, pinching, and pressing—were used to perceive the stimuli. In Experiment 1 (without a contextual task), participants estimated the perceived intensity of softness or pleasantness for each stimulus. In Experiment 2 (with a contextual task), the participants sequentially perceived two stimuli with different compliance levels and indicated which stimulus they perceived as softer and pleasant. The results showed that the psychophysical relationship between compliance and perceived softness was consistent across all tactile strategies in both experiments, with softness estimates increasing as compliance increased. However, the relationship between compliance and pleasantness differed between the two experiments. In Experiment 1, pleasantness estimates increased monotonically with increased compliance. However, in Experiment 2, across all tactile strategies, pleasantness began to decrease within the compliance range of 0.25–2.0 cm^2^/N, exhibiting an inverted U-shaped trend. These findings indicate that the relationship between compliance and pleasantness is task-dependent, particularly demonstrating significantly different trends when a contextual task is introduced. In contrast, the relationship between compliance and softness remained consistently monotonic.

## 1. Introduction

When we touch objects, we can perceive and distinguish their physical properties while simultaneously experiencing related emotional responses, such as feelings of pleasantness or unpleasantness. In psychophysical research, most studies have focused on discriminative touch, exploring the relationships among sensory perception, physical attributes and variations in stimuli (LaMotte, 2000; Srinivasan & LaMotte, 1995). Given the pivotal role of emotional touch in quality of life and overall well-being, there has been a surge of interest in elucidating the underlying mechanisms of affective touch (Löken et al., 2011; McGlone et al., 2014). In previous studies, the impact of physical properties related to discriminative touch on pleasure has been examined, and psychophysical functions related to softness and pleasantness have been explored under both active (Kitada et al., 2021) and passive (Pasqualotto et al., 2020) pressing conditions. However, the relationship between softness and pleasantness when other touch strategies are used remains unclear. Additionally, the pleasure derived from touch is influenced not only by stimulus-driven processes but also by the specific contextual task in which the touch occurs (Ellingsen et al., 2016). This study initially examines the relationship between softness and pleasure across a range of touch strategies and investigates the effects of the presence and absence of contextual tasks on this relationship.

Softness, as a material property, is a primary dimension of discriminative touch and has been shown to be correlated with tactile pleasantness (Kitada et al., 2021). It is associated with the compliance of an object, which is determined by the material’s elasticity. The degree of elasticity is typically quantified by Young’s modulus (*E*), which is defined as the ratio of stress to strain. Furthermore, compliance can be expressed in terms of stiffness, which is defined as the ratio of force to displacement. In contrast to the influence of the Young’s modulus, the property of stiffness is subject to additional factors, including the thickness of the object and the dimensions of the contact area (Bergmann Tiest & Kappers, 2009). Consequently, stiffness is not an intrinsic material property; rather, it depends on the specific object and the touch strategy employed. This indicates that stiffness-based compliance places constraints on the range of touch strategies that can be employed. In this study, we defined the physical measure of stimulus compliance as the reciprocal of Young’s modulus, thereby ensuring its independence from the object’s shape and size.

In active touch, humans deploy a range of tactile strategies, contingent on their size, shape, and exploration purpose, to perceive the softness of objects (Cavdan et al., 2021; Drewing, 2014; Lederman & Klatzky, 1987). The most common touch strategy involves pressing the fingertip against a surface, pinching the object between the thumb and index finger, or grasping it with the entire palm. These various touch strategies modify tactile and kinesthetic information, including both proprioceptive and cutaneous cues (Friedman et al., 2008). Both types of signals offer essential sensory information for accurately perceiving an object’s softness. Therefore, although different touch strategies alter the perception of an object’s softness (Xu et al., 2019), it remains unclear whether they significantly affect tactile pleasantness. To explore the influence of touch strategies on pleasure perception and compare their relationship with softness, this study designed two differently shaped stimuli with identical compliance. On the basis of three commonly used touch strategies in daily life, this study investigated the impact of these strategies on pleasantness estimation.

Moreover, tactile pleasantness is influenced not only by stimulus-driven processes but also by top-down cognitive mechanisms, such as expectations and prior knowledge. Human estimation of tactile pleasantness is based on the brain’s integration of tactile information and prior experiences (Gallace & Spence, 2010). This has been extensively studied in the context of social touch, where specific tactile actions can convey the emotional state of the person initiating the touch to the recipient (Hertenstein et al., 2009; Kirsch, 2018). Positive visual content increases the estimation of tactile pleasantness, whereas negative visual content decreases it (Etzi et al., 2018; Ravaja et al., 2017). The mere exposure effect has also been observed in affective tactile modalities (Jakesch & Carbon, 2012). This evidence indicates that the contextual environment plays a significant role in generating tactile pleasantness. However, it remains unclear whether prior information influences the psychophysical relationship between an object’s softness and the resulting pleasantness. To better understand the nature of tactile pleasantness, it is essential to compare pleasantness estimates across various contextual tasks.

In summary, we conducted two experiments to examine whether touch strategies modify the psychophysical relationship between softness and pleasantness and whether contextual tasks influence this relationship. This is crucial for uncovering the relationship between the sensory mechanisms underlying discriminative touch and affective touch. In Experiment 1, the participants were asked to actively assess the softness and pleasantness of various compliant stimuli via three distinct touch strategies. They provided numerical estimates of the perceived magnitude of softness and pleasantness. This experiment explored the psychophysical relationship between softness and pleasantness across different touch strategies. In Experiment 2, we employed a two-alternative forced choice (2AFC) method, where participants selected the softer or more pleasant of two sequentially presented stimuli. We argue that when two stimuli are compared, the psychophysical models of softness and pleasantness from the two experiments differ if the perception of the first stimulus affects the perceived softness or pleasantness of the second stimulus.

## 2. Experiment 1: Softness and Pleasantness Estimation Under Three Different Touch Strategies

### 2.1. Materials and Methods

#### 2.1.1. Participants

The study recruited 30 right-handed male participants (20–33 years), who were divided into two groups of 15 based on age. The first group performed a pleasantness estimation task, whereas the second group performed a softness estimation task. This was performed to avoid gender-related differences in affective tactile processing (Schirmer et al., 2022) and to prevent participants from reporting softness as pleasantness. The participants received research participation credits or JPY 1000 for 60 min. All participants had uninjured fingers and palms and had no history of mental or neurological disorders. Handedness was determined on the basis of the Fazio Handedness Scale. 

All the participants were naive to the purpose of the ongoing experiment. All participants signed written informed consent forms in line with the guidelines of the Medical Ethics Committee at Okayama University. The committee also reviewed and approved the testing procedures.

To ensure sufficient statistical power, we conducted a post hoc simulation-based power analysis on the results from the two groups in Experiment 1 via the mixedpower package in R (version 4.3.1) (Kumle et al., 2021). Given that post hoc procedures can overestimate the true effect size, we conducted the simulations using half of the observed effect size. The results indicated that a sample size of 15 participants per group yielded a power exceeding 80%.

#### 2.1.2. Stimuli and Touch Strategies

**Figure 1:**
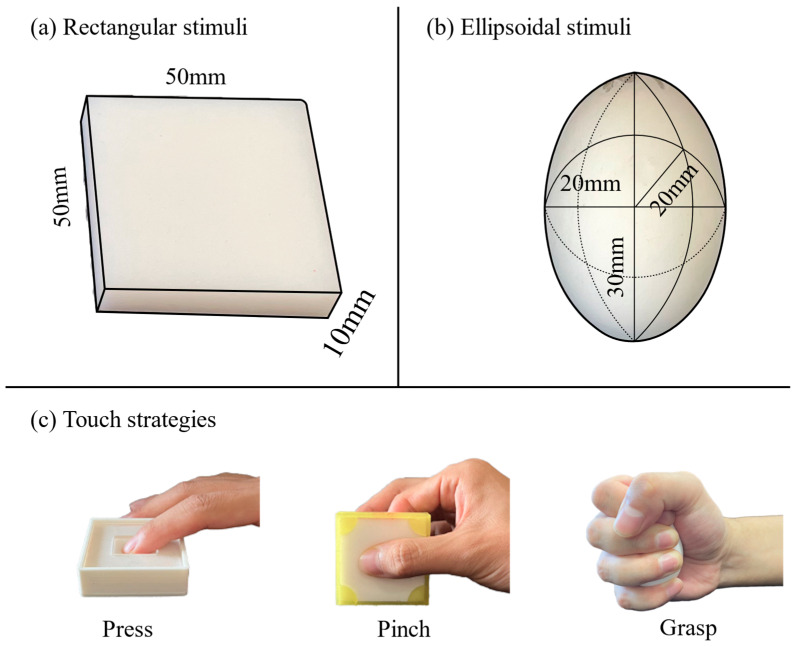
The two different stimulus shapes and the three touch strategies. (**a**) Eleven rectangular stimuli were made from silicone rubber, with baby powder applied evenly on the surface to minimize tackiness. (**b**) Eleven ellipsoidal stimuli were made from silicone rubber. The Young’s modulus values of these stimuli were the same as those of the rectangular stimuli. (**c**) Press task: The participants used their right index finger to press the center of each stimulus. After pressing the surface twice, the participants were asked to estimate the softness or pleasantness of the stimulus. Pinch task: The participants pinched the center of each stimulus between their right index finger and thumb. Grasp task: The participants grasped each stimulus in the palm of their right hand.

We constructed twenty-two stimuli using two different shapes (Figure 1a,b), rectangular and ellipsoidal, from noncarcinogenic silicone rubber (TANAC Co. Ltd., Gifu, Japan). The dimensions of the rectangular stimuli were 50 mm × 50 mm × 10 mm, and the ellipsoidal stimuli had radii of 30 mm for the long axis and 20 mm for the two other axes. The compliance was identical for both stimuli, with 11 levels for each (Table 1). The compliance of each stimulus was calculated as the inverse of Young’s modulus, which was measured for each stimulus via the same instrument (SOFTGRAM, SHINKO DENSHI Co., Ltd., Tokyo, Japan). The error in the Young’s modulus of each stimulus ranged from 5% to 10%. Each stimulus was sufficiently coated with baby talcum powder to prevent a sticky sensation.

The rectangular stimulus was used for pressing and pinching, whereas the ellipsoidal stimulus was used for grasping (Figure 1c). This is determined by the way interactions with the stimuli are conducted during active touch. When participants pressed or pinched the rectangular stimuli, they applied force to the surface, resulting in surface indentation. In this scenario, the applied and reactive forces were perpendicular. However, when participants grasped the rectangular object, the entire stimulus not only experienced indentation but also bent, generating additional sensory information. The ellipsoidal stimulus avoided this issue, as its shape allowed the fingers and palm to evenly envelop the object, ensuring that the applied and reactive forces were distributed along the contact surface without inducing significant bending or additional sensory distortion.

#### 2.1.3. Experiment 1 Design

The experiment consisted of two within-subject factors and one between-subject factor. Compliance and touch strategy were within-subject factors, whereas perception (softness and pleasantness) was the between-subject factor used to prevent participants from equating pleasantness with softness. To avoid fatigue, the experiment was conducted over three days, with three sessions. In each session, the participants were instructed to use one touch strategy, with three different touch strategies applied in a pseudo-random manner across the three sessions. The order of the 11 compliance stimuli was pseudorandomized for each participant’s 10 repetitions to ensure that each stimulus appeared twice in each repetition. Before the experiment, the participants completed at least 22 practice trials to gain a general understanding of the range of stimulus compliance, which were excluded from the statistical analysis.

#### 2.1.4. Experiment 1 Procedure

**Figure 2:**
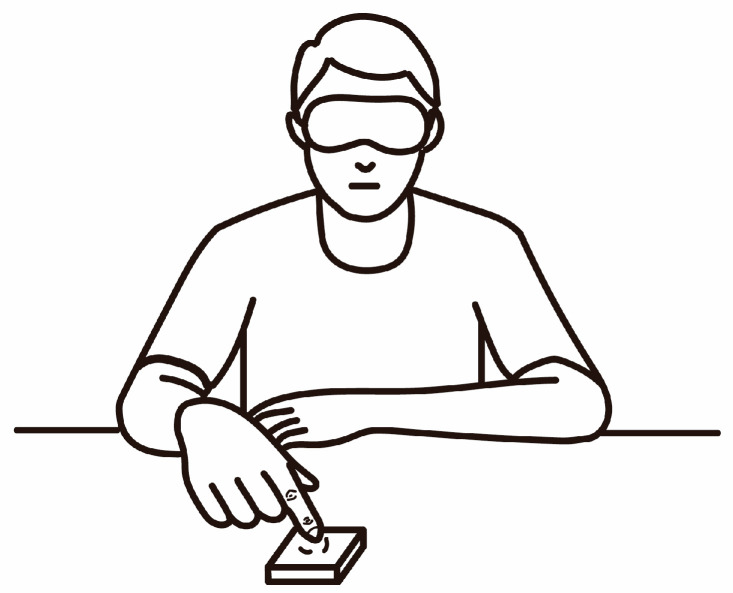
Experimental scene. As an example of the pressing strategy, participants sat blindfolded at a table with the stimulus positioned to their right. They used the index finger of their right hand to press the stimulus.

The participants were seated in height-adjustable chairs with the compliance stimulus on their right side (Figure 2). We used an absolute magnitude estimation procedure to estimate the softness and pleasantness of each compliant stimulus. In the softness group, the participants were asked to choose a number corresponding to perceived softness. In the pleasantness group, the participants were asked to choose a number corresponding to the stimulus-related pleasantness. To ensure that softness was not confused with pleasantness, the experimental instructions for the pleasantness group explicitly excluded any terms related to softness. The participants were asked to choose any number from 1 to 100, with higher numbers indicating greater softness or greater pleasantness evoked by the stimulus. No standards were used. All the participants were blindfolded.

Before the experiment began, the experimenter, seated on the opposite side of the table, instructed the participant to use the designated touch strategy to explore the stimuli and ensured that the participant knew the location of the next stimulus. For the ellipsoidal stimulus, participants opened their right hand, and the experimenter placed the stimulus in their palm. The participants were allowed to touch the stimulus up to two times. After each trial, the participants verbally reported their estimated value, which the experimenter recorded. A 2 min break was given after every 22 trials. Before the experiment, the participants were instructed to explore each stimulus via a natural amount of force. Each session lasted 60 min, resulting in a total session duration of 180 min for experiment 1.

### 2.2. Results and Discussion

All mean estimates were transformed via a log function (base 10), and the transformed data were used as the basis for subsequent analyses. Owing to the presence of variance differences between the stimuli (Levene’s test) and the fact that pleasantness, as a subjective sensation, is influenced by individual preferences (Kitada et al., 2021). Therefore, we employed a linear mixed model (LMM) for data analysis, via the lme4 package in R (version 4.3.1) (Bates et al., 2015). The LMM approach allows for simultaneous handling of both fixed and random effects. By treating individual preferences as random effects, we were able to account for individual variability, resulting in more precise estimates of the fixed effects.

Separate models were constructed for the estimates of the two groups, incorporating the same fixed factors: compliance, touch strategy, and their interaction. To mitigate the issue of multicollinearity, the continuous fixed factor, compliance, was standardized into z scores. Type III ANOVA F-statistics were reported, and significant interactions were followed by Bonferroni-corrected post hoc pairwise comparisons.

The random effects were determined via model comparisons using likelihood ratio tests. The participants were treated as random intercepts, and random slopes for the significant fixed factors of interest were progressively fitted. When the model comparison indicated a significant improvement, more complex random structures were employed (Baayen et al., 2008). In the mean pleasantness model, the random slope for compliance across participants was significant (*p* < 0.001). In the mean softness model, the random slope for the touch strategy across participants was significant (*p* < 0.001). Models incorporating the remaining fixed factors as random slopes yielded insignificant results or failed to converge. All data processing and analyses were performed in R (4.3.1).

#### 2.2.1. The Impact of Compliance and Touch Strategy

Pleasantness and softness are two distinct perceptual dimensions that are assessed by two separate groups of participants. To standardize the estimation of the quantitative values, we specified the range from 1 to 100. However, we were unable to directly compare the quantitative evaluations of these two perceptions. As a result, we analyzed the data from each perceptual dimension separately to examine the effects of different touch strategies and stimulus compliance.

**Figure 3:**
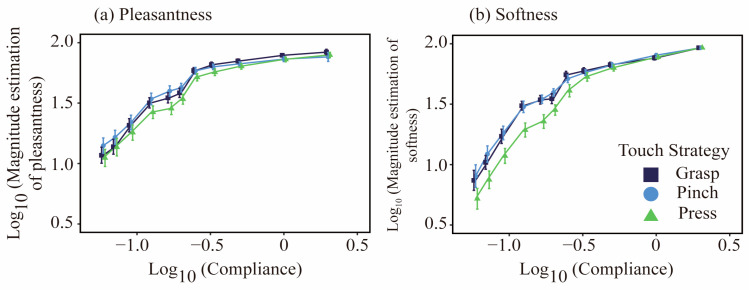
Mean log_10 magnitude estimates of pleasantness and softness as a function of compliance for three touch strategies. Each data point indicates the mean ± SEM.

- Pleasantness:

Figure 3a shows the relationship between pleasantness and object compliance on a base 10 logarithmic scale. The mean pleasantness increased monotonically with increasing compliance across the three touch strategy conditions but plateaued at a compliance value of 0.25 ${\mathrm{cm}}^{2}/N$ (−0.602 on the base 10 logarithmic scale). This finding indicates that beyond a certain level of compliance, further increases in an object’s compliance lead to diminishing returns in perceived pleasantness. The patterns of perceived pleasantness and softness were consistent across the three touch strategies, although there were variations in sensitivity.

Statistical analyses revealed significant main effects for compliance ($F\left(1,14\right)=177.1,\;p<0.001$), whereas the main effect of touch strategy was not significant ($F\left(2,461\right)=\;1.41,\;p=0.24$). Additionally, the interaction between the two factors was significant ($F\left(2,461\right)=3.56,\;p=0.029$). Bonferroni-corrected post hoc pairwise comparisons indicated that pleasantness estimates were lower under the press strategy than under the other two touch strategies. Within the compliance range of 0.059 to 0.20 ${\mathrm{cm}}^{2}/N$, the pleasantness estimates under the press strategy were significantly lower than those under the pinch strategy (see Table 2).

- Softness:

Figure 3b shows the relationship between perceived softness and object compliance on a base 10 logarithmic scale. Similarly to the pleasantness estimates, the mean softness estimates increased monotonically with compliance across the three touch strategy conditions. Statistical analysis revealed a significant main effect of compliance ($F\left(1,447\right)=1398.6,\;p<0.001$), whereas the main effect of touch strategy was not significant ($F\left(2,447\right)=\;0.11,\;p=0.89$). Moreover, there was a significant interaction effect between compliance and touch strategy ($F\left(2,447\right)=6.8,\;p=0.0013$). Table 3 presents the results of the post hoc pairwise comparisons adjusted via the Bonferroni correction. Consistent with the results for pleasantness, within the compliance range of 0.059 to 0.20 ${\mathrm{cm}}^{2}/N$, the softness estimates under the press strategy were significantly lower than those under the pinch strategy.

#### 2.2.2. Variances of Magnitude Estimates

**Figure 4:**
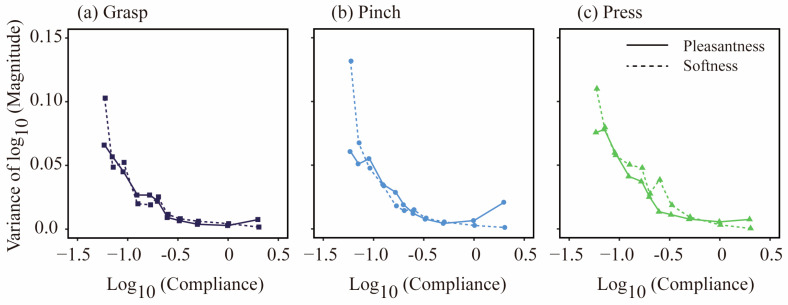
Variance in magnitude estimates. Variances of ${log}_{10}$ magnitude estimates were plotted as a function of $\log_{10}$ compliance.

As shown in Figure 3, the standard errors of the means varied significantly across compliance levels. We analyzed the variance in magnitude estimates via Levens’ test to explore individual differences across the two perceptual dimensions. Figure 4 illustrates the relationship between variance and compliance. While the variance in pleasantness showed an upward trend toward the higher end of compliance (2.0 ${\mathrm{cm}}^{2}/N$), the variance in softness decreased monotonically with increasing compliance.

To statistically evaluate the different variance patterns among stimuli, we conducted Levene’s chi-square test to statistically assess differences in variance across compliance levels. The variance of stimuli that elicited high levels of pleasantness under the three touch strategies was significantly smaller than that of lower compliance stimuli. Similarly, the same test of softness revealed that the variance for low-compliance stimuli was significantly greater than that for high-compliance stimuli (*p* values < 0.05).

In summary, consistent with prior research, compliance plays a critical role in determining tactile pleasantness. The patterns of perceived pleasantness and softness were consistent across the three touch strategies, although there were variations in sensitivity. However, it remains unclear whether the relationship between affective and discriminative touch changes with task context. We hypothesize that perceived softness is influenced primarily by the physical intensity of the stimulus, whereas contextual tasks have a greater impact on tactile pleasantness. Therefore, in Experiment 2, we introduced a contextual task to explore the relationship between softness and pleasantness.

## 3. Experiment 2: Softness and Pleasantness Discrimination Experiment

### 3.1. Materials and Methods

#### 3.1.1. Participants and Stimuli

Fifteen right-handed participants (all male) were recruited for Experiment 2. No participants had completed Experiment 1. All the participants completed the softness and pleasantness discrimination tasks; all the participants first completed the pleasantness discrimination task, followed by the softness discrimination task. The participants received JPY 1000 or research participation points every 60 min. All participants had uninjured fingers and palms and had no history of mental or neurological disorders. Handedness was determined on the basis of the Fazio Handedness Scale. All the participants were naive to the purpose of the ongoing experiment. All participants signed written informed consent forms in line with the guidelines of the Medical Ethics Committee at Okayama University. The committee also reviewed and approved the testing procedures.

The stimuli and touch strategies were identical to those in Experiment 1.

#### 3.1.2. Design and Procedure

In Experiment 2, we employed the two-alternative forced choice method (2-AFC). The participants were instructed to sequentially touch two stimuli with different levels of compliance and judge whether the second stimulus felt softer or more pleasurable on the basis of the first stimulus. To minimize the potential influence of repeated exposure to a single standard stimulus on preference levels (Jakesch & Carbon, 2012), we used four different standard stimuli, each with different compliance levels (see Table 4).

The experiment included three within-subject factors: perception (pleasantness and softness), touch strategy, and stimulus combination (four standard stimuli with different compliance levels, each paired with ten comparison stimuli). Each trial condition was repeated 10 times. Each participant completed a total of 2400 trials, which were conducted over 12 sessions within two months. The first six sessions for all participants focused on pleasantness discrimination, whereas the latter six sessions were dedicated to softness discrimination. The touch strategy was changed every two sessions, with the sequence determined pseudorandomly. The selection of stimulus combinations was also determined pseudorandomly by the standard stimulus, with all trials involving two standard stimuli completed within each session. The probability of the standard stimulus appearing first in these trials was 50%. Each session lasted 90 min, resulting in a total session duration of 1080 min for Experiment 2. The experimental setup was the same as that in Experiment 1. The participants verbally reported which of the two stimuli was softer or more pleasant, and the responses were recorded by the experimenter.

### 3.2. Results and Discussion

In Experiment 2, the selection rate of the comparison stimulus was used as the dependent variable, and when it exceeded 50%, it could be interpreted as the comparison stimulus being more pleasant than the standard stimulus. Notably, different standard stimuli influenced the selection rate of the same compliance comparison stimuli. However, this was not the focus of the present study, which aimed to investigate whether the relationship between tactile pleasantness and compliance changes in the presence of a contextual task.

Additionally, similar to the results of Experiment 1, owing to the variance between the stimuli, we used generalized linear mixed models (GLMMs) to model the data, with the selection of the comparison stimulus (binomial link function) as the dependent variable. The purpose of this study was to analyze whether the relationship between pleasantness and compliance under a contextual task is affected by the touch strategy. We constructed separate models for the results of each standard stimulus, with participants included as random intercepts.

The method for determining random effects was the same as that in Experiment 1. The participants were included as random intercepts, whereas the touch strategy and comparison stimulus compliance were included as random slopes (GLMM model: ~compliance × touch strategy + (1 + touch strategy + compliance | participant)). The following section provides detailed information on the fixed effects of the touch strategy on the basis of likelihood ratio tests.

- Pleasantness

**Figure 5:**
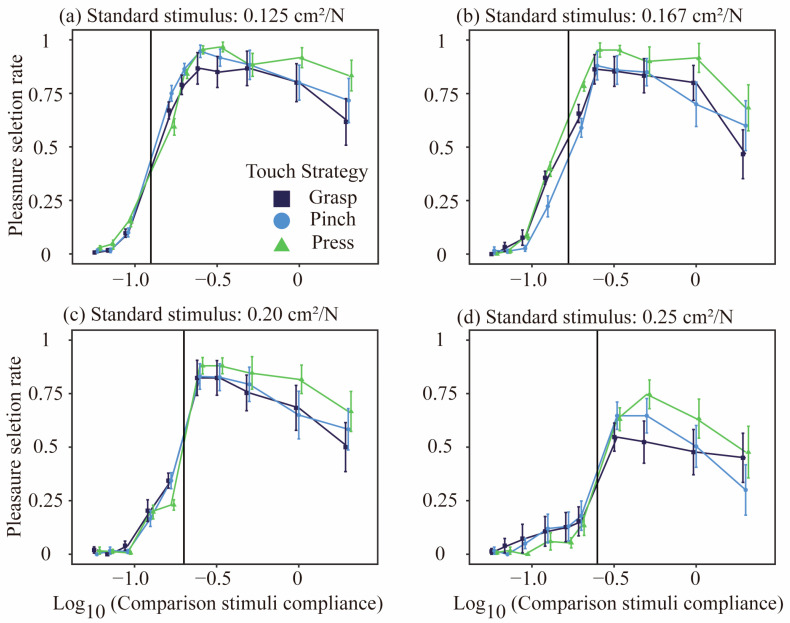
Pleasure selection rates as a function of comparison stimulus compliance across four standard stimuli with different compliance levels: (**a**) 0.125 ${\mathrm{cm}}^{2}/N$, (**b**) 0.167 ${\mathrm{cm}}^{2}/N$, (**c**) 0.20 ${\mathrm{cm}}^{2}/N$, and (**d**) 0.25 ${\mathrm{cm}}^{2}/N$. The x-axis represents the logarithm (base 10) of the comparison stimulus compliance, whereas the y-axis represents the pleasure selection rate. Three touch strategies—Grasp (purple), Pinch (blue), and Press (green)—were evaluated via the two-alternative forced choice method. Each data point corresponds to the mean pleasure selection rate, with error bars indicating the standard error (SE). The black lines represent the compliance levels of the standard stimuli used in each condition.

Figure 5 shows the relationship between the compliance with the comparison stimulus and the pleasantness selection rate across the four standard stimuli. At all four levels, the selection rate increased monotonically as the compliance of the comparison stimulus remained below that of the standard stimulus. However, when compliance exceeded that of the standard stimulus, the selection rate rose to a peak and then began to decline, forming an overall inverted U-shaped trend. Additionally, compliance with the standard stimulus affected the difficulty of pleasantness discrimination; as compliance increased, the peak selection rate decreased, making the pleasantness discrimination task more challenging.

For each standard stimulus condition, we evaluated whether the touch strategy influenced the selection rate of the comparison stimuli. Touch strategies with three levels (grasp, pinch, and press) were included as a fixed-effect variable in the model and compared to a model that excluded touch strategies. The touch strategy significantly influenced the selectivity of the comparison stimulus at a standard stimulus compliance of 0.167 ${\mathrm{cm}}^{2}/N$ (logLik with touch strategy = −3918.4, $\chi^{2}\left(2\right)=\;7.5292,\;p=0.023$). No significant changes were observed in the other standard stimulus conditions ($0.125\;{\mathrm{cm}}^{2}/N$: logLik = −3714.1, $\chi^{2}\left(2\right)=\;2.3071,\;p=0.316$; 0.20 ${\mathrm{cm}}^{2}/N$: logLik = −3942.8, $\chi^{2}\left(2\right)=\;3.0539,p=0.217$; 0.25 ${\mathrm{cm}}^{2}/N$: logLik = −3374.3, $\chi^{2}\left(2\right)=1.3925,\;p=0.499$).

- Softness

**Figure 6:**
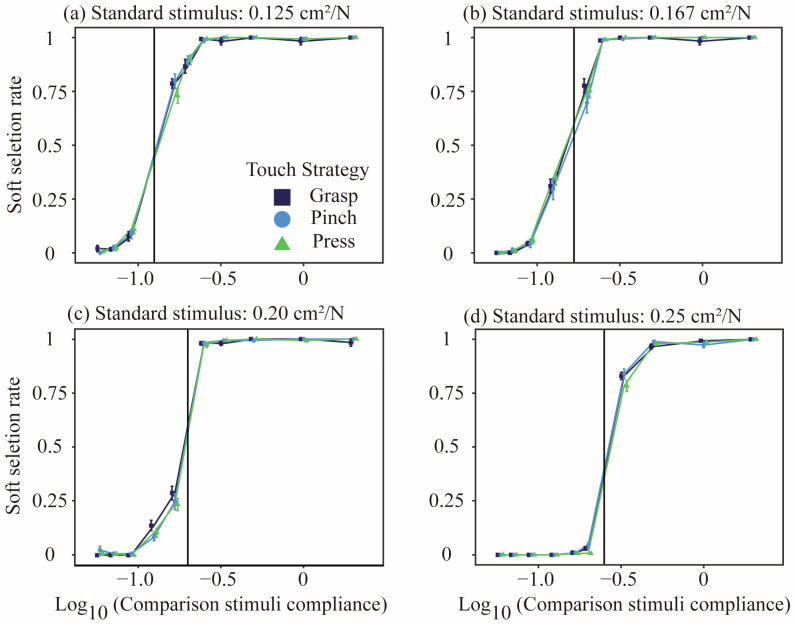
Soft selection rates as a function of comparison stimulus compliance across four standard stimuli with different compliance levels: (**a**) 0.125 ${\mathrm{cm}}^{2}/N$, (**b**) 0.167 ${\mathrm{cm}}^{2}/N$, (**c**) 0.20 ${\mathrm{cm}}^{2}/N$, and (**d**) 0.25 ${\mathrm{cm}}^{2}/N$. The x-axis represents the logarithm (base 10) of the comparison stimulus compliance, whereas the y-axis represents the soft selection rate. Three touch strategies—Grasp (purple), Pinch (blue), and Press (green)—were evaluated via the two-alternative forced choice method. Each data point corresponds to the mean pleasure selection rate, with error bars indicating the standard error (SE). The black lines represent the compliance levels of the standard stimuli used in each condition. The black lines represent the compliance levels of the standard stimuli used in each condition.

Figure 6 shows the relationship between compliance with the comparison stimulus and the pleasantness selection rate across the four standard stimuli. At all four levels, the relationship between the compliance of the comparison stimulus and the selection rate presented an S-shaped curve, indicating that the compliance of the object determined the ability to discriminate softness. Softness selectivity was analyzed via the same statistical approach as for pleasantness, and no significant differences in touch strategy were found across any of the standard stimulus conditions (0.125 ${\mathrm{cm}}^{2}/N$: logLik with touch strategy = −1402.1, $\chi^{2}\left(2\right)=0.9879,\;p=0.610$; 0.167 ${\mathrm{cm}}^{2}/N$: logLik = −1466.0, $\chi^{2}\left(2\right)=\;4.6588,\;p=0.098$; 0.20 ${\mathrm{cm}}^{2}/N$: logLik = −1274.0, $\chi^{2}\left(2\right)=1.1477,\;p=0.563$; 0.25 ${\mathrm{cm}}^{2}/N$: logLik = −834.71, $\chi^{2}\left(2\right)=5.1997,\;p=0.074$).

The participants demonstrated different patterns in the softness and pleasantness models across the three touch strategies during the discrimination task. Softness discrimination was entirely dependent on the magnitude of the object’s compliance, whereas pleasantness discrimination displayed an inverted U-shaped curve when the compliance of the comparison stimulus exceeded that of the standard stimulus. The results indicate that the softness estimation is entirely dependent on the degree of deformation of the object under the applied force, whereas this was not the case for the pleasantness estimation.

## 4. Discussion

This study investigated the estimation of pleasantness and softness elicited by three different touch strategies when individuals interact with objects of varying compliance via two distinct psychophysical experimental paradigms. Additionally, the impact of a contextual task on both pleasantness and softness was assessed by comparing the results from the two experiments.

### 4.1. The Impact of a Contextual Task on the Estimation of Pleasantness and Softness

The psychophysical relationships between pleasantness and softness in the context-free task (magnitude estimation experiment) varied similarly with object compliance, with participants perceiving greater pleasantness and softness as the object’s compliance increased. When a contextual task was introduced (discrimination experiment), peak pleasantness did not correspond to the most compliant stimuli; instead, it followed an inverted U-shaped curve. However, the estimation of softness remained unchanged.

Softness is a subjective measure of an object’s compliance, reflecting its ability to deform under pressure (Di Luca & Ernst, 2014). Regardless of the task context, the softness model remains consistent, increasing monotonically with the object’s compliance (Friedman et al., 2008). Humans perceive the softness of objects by integrating sensory information from musculomotor signals at the joints and cutaneous mechanoreceptors (Metzger & Drewing, 2015; Scilingo et al., 2010; Srinivasan & LaMotte, 1995). The musculomotor signals provide feedback on the finger position and applied force, whereas the cutaneous mechanoreceptors convey information about the spatial distribution of pressure within the contact area between the finger and the stimulus. Humans exhibit perceptual constancy for physical properties, enabling relatively stable and realistic perceptions. However, this constancy is diminished regarding pleasantness sensations. For example, both deep pressure massage (Mullen et al., 2008) and light touch (Löken et al., 2009) can evoke pleasantness responses. This is because the experience of pleasantness is not solely driven by external stimuli but is also influenced by motivation and internal perception. As a psychological feeling, pleasantness is influenced by numerous factors, including individual emotions, cognitive states, and the external environment (Russell & Mehrabian, 1978; Smith & Ellsworth, 1985). Thus, the two experimental paradigms influenced the pleasantness model through differing task requirements and task contexts.

Specifically, the experimental paradigm altered the criteria by which participants perceived pleasantness. In estimation experiments, participants are required to assign a value to the pleasantness of each varying level of compliance, which requires not only processing sensory information but also integrating this information to form an explicit quantitative judgment. This task is more complex and cognitively demanding. Participants must establish an internal reference among all stimuli, and the formation of such a scale often depends on everyday life experiences. Soft surfaces are frequently linked to positive experiences (Drewing et al., 2018; Guest et al., 2011), leading participants to associate softness with pleasantness on the basis of this long-established perceptual model. Relatively speaking, discrimination experiments are less cognitively demanding, as participants need to compare only the intensity of pleasurable sensations between two objects. This requires processing less information, and participants are not required to establish complex internal scales. Instead, they can make relative judgments on the basis of immediate perceptions.

From a neurophysiological perspective, the perception of softness primarily involves processing in the primary somatosensory cortex (S1) and secondary somatosensory cortex (S2), which are responsible for processing tactile information (Kitada et al., 2019). In contrast, the perception of pleasantness involves a wider network of brain regions, including the limbic system (such as the nucleus accumbens and amygdala) (Gothard & Fuglevand, 2022) and multimodal integration areas (such as the insula and anterior cingulate cortex) (Rolls, 2003). Additionally, the activity patterns in these brain regions can also change with task context. For example, during quantity estimation tasks, brain regions involved in numerical and value assessment, such as the parietal cortex, may become more active (Shuman & Kanwisher, 2004). Conversely, in discrimination tasks, regions associated with emotional evaluation and decision-making, such as the ventromedial prefrontal cortex, may play a more significant role (Alexander et al., 2023; Motzkin et al., 2015).

In summary, this study provides important insights into the relationship between the two perceptual dimensions of the tactile system. Previous psychophysical research approaches have largely focused on examining the influence of a few key parameters on how discriminative haptics affect emotional perception (Kitada et al., 2012). However, the findings of this study highlight that affective haptics involve complex intrinsic mechanisms that cannot be fully revealed through simple parameter manipulations or comparisons with discriminative haptics. Instead, it is crucial to align research objectives with carefully designed experimental paradigms to better understand the unique functions and underlying mechanisms of affective haptics.

### 4.2. The Impact of Touch Strategies in the Two Experiments

The impacts of the touch strategy on the softness and pleasantness estimations were nearly identical. In the estimation experiments, no significant differences were observed between the grasp and pinch strategies, except for a difference in pleasantness estimates under the 0.059 ${\mathrm{cm}}^{2}/N$ compliance condition. The impact of touch strategy is most evident in the comparison between the press and the other two touch strategies (Table 2). In the discrimination experiments, touch strategies differed only in the pleasantness discrimination task with a standard stimulus of 0.167 ${\mathrm{cm}}^{2}/N$, whereas no significant differences were observed in the other tasks.

The differences in touch strategy were reflected primarily in lower estimates of pleasantness and softness for the press strategy. The spatial distributions of skin deformation and pressure provide crucial information for assessing compliance (Li et al., 2023). When estimating objects with deformable surfaces, humans tend to move their fingers to specific displacements to induce variations in reaction forces, thereby obtaining relevant information about compliance (Xu et al., 2019). Upon contacting an object, the initial finger displacement is relatively large, whereas the reaction force is small. As the displacement increases, the reaction force gradually increases. The brain automatically adjusts the strength of contact and the positioning of the fingers to obtain more accurate information when it detects changes in force (Kaim & Drewing, 2011). Compared with the other two strategies, the pressing strategy generates smaller forces, as all the applied and reactive forces are borne solely by the muscles and joints of the index finger. When the object has low compliance (harder), greater pressing force is needed to create sufficient indentation and obtain adequate sensory information. In the pressing strategy, the finger joints experience higher localized pressure, as only the muscles and joints of the index finger supply the force. This results in a perception of increased hardness. Pleasantness is intricately tied to the perception of softness (Kitada et al., 2021; Pasqualotto et al., 2020). In discrimination experiments, participants do not need to fully perceive the compliance of the stimulus but rather compare the relative differences between two stimuli. All three touch strategies demonstrated a high level of accuracy in terms of discrimination compliance.

Additionally, the three touch strategies examined in this study are based on interactions with compliant objects under the exploratory strategy pressure. Recent studies, however, have demonstrated that softness encompasses multiple dimensions, such as furriness and granularity (Dövencioğlu & Drewing, 2018), which influence the perception of softness. Moreover, different touch strategies may be associated with various dimensions of softness (Cavdan et al., 2021). Therefore, additional research is needed to investigate whether the relationship between softness and pleasantness remains consistent across these different dimensions and touch strategies.

## 5. Conclusions

In summary, we investigated how humans use three different touch strategies to perceive the softness and pleasantness of objects across various contextual tasks. Through estimation experiments, we first established the psychophysical relationship between softness and pleasantness for three touch strategies, confirming that softness plays a key role in influencing tactile pleasantness. In the discrimination experiments, we observed that the functional relationship between softness and pleasantness, as identified in the estimation experiments, shifted when a contextual task was introduced. This demonstrated the significant impact of contextual tasks on the estimation of tactile pleasantness.

From a theoretical perspective, this study deepens our understanding of the multi-level processing mechanisms of tactile perception, and, in particular, provides new evidence on the interaction between affective and discriminative touch. From an application perspective, the findings highlight the importance of focusing not only on the basic physical properties of tactile stimuli, but also fully considering the task context, top-down cognitive factors, and the diversity of tactile strategies when designing experiments or applications (e.g., haptic interfaces). By integrating these factors, future research will have a better chance of unraveling the complex mechanisms of affective and discriminative haptics.

## Figures and Tables

**Table 1:** Stimulus shape and compliance.

Shape	$\mathrm{Compliance}\;({\mathrm{cm}}^{2}/N)$
Rectangular	0.059, 0.071, 0.091, 0.125, 0.167, 0.2, 0.25, 0.33, 0.5, 1.0, 2.0
Ellipsoidal	0.059, 0.071, 0.091, 0.125, 0.167, 0.2, 0.25, 0.33, 0.5, 1.0, 2.0

**Table 2:** Post hoc pairwise comparisons of the pleasantness estimate.

Model Formula:	log_10_ (Estimated Pleasantness)~Compliance × Touch Strategy + (1 + Compliance|Participant)					
Compliance	Touch Strategy					
$({cm}^{2}/N$)	Grasp–Pinch		Grasp–Press		Pinch–Press	
	Estimate (SE)	*p*	Estimate (SE)	*p*	Estimate (SE)	*p*
0.059	−0.07 (0.027)	1.0	0.05 (0.027)	1.0	0.12 (0.027)	*
0.071	−0.06 (0.025)	1.0	0.05 (0.025)	1.0	0.11 (0.025)	**
0.091	−0.05 (0.022)	1.0	0.05 (0.022)	1.0	0.10 (0.022)	**
0.125	−0.04 (0.019)	1.0	0.05 (0.019)	1.0	0.09 (0.019)	**
0.167	−0.03 (0.017)	1.0	0.05 (0.017)	1.0	0.08 (0.017)	**
0.200	−0.02 (0.017)	1.0	0.05 (0.017)	1.0	0.07 (0.017)	*
0.250	−0.02 (0.016)	1.0	0.05 (0.016)	1.0	0.06 (0.016)	0.07
0.333	−0.01 (0.017)	1.0	0.05 (0.017)	1.0	0.05 (0.017)	1.0
0.500	0.001 (0.020)	1.0	0.05 (0.020)	1.0	0.04 (0.020)	1.0
1.000	0.03 (0.028)	1.0	0.05 (0.028)	1.0	0.01 (0.028)	1.0
2.000	0.06 (0.037)	1.0	0.04 (0.037)	1.0	−0.01 (0.037)	1.0

* *p* value < 0.05and ** *p* value < 0.01.

**Table 3:** Post hoc pairwise comparisons of the softness estimate.

Model Formula:	log_10_ (Estimated Pleasantness)~Compliance × Touch Strategy + (1 + Touch strategy|Participant)					
Compliance	Touch Strategy					
$({cm}^{2}/N$)	Grasp–Pinch		Grasp–Press		Pinch–Press	
	Estimate (SE)	*p*	Estimate (SE)	*p*	Estimate (SE)	*p*
0.059	−0.03 (0.049)	1.0	0.18 (0.052)	0.70	0.21 (0.037)	***
0.071	−0.03 (0.047)	1.0	0.17 (0.050)	0.99	0.20 (0.034)	***
0.091	−0.02 (0.045)	1.0	0.16 (0.048)	1.0	0.18 (0.031)	***
0.125	−0.02 (0.042)	1.0	0.14 (0.045)	1.0	0.16 (0.027)	**
0.167	−0.02 (0.041)	1.0	0.12 (0.044)	1.0	0.14 (0.025)	*
0.200	−0.02 (0.041)	1.0	0.11 (0.044)	1.0	0.13 (0.024)	*
0.250	−0.01 (0.040)	1.0	0.10 (0.044)	1.0	0.11 (0.024)	0.15
0.333	−0.01 (0.041)	1.0	0.08 (0.044)	1.0	0.09 (0.025)	0.75
0.500	−0.005 (0.04)	1.0	0.06 (0.046)	1.0	0.06 (0.028)	1.0
1.000	0.003 (0.050)	1.0	0.02 (0.053)	1.0	0.02 (0.038)	1.0
2.000	0.01 (0.059)	1.0	−0.02 (0.062)	1.0	−0.03 (0.050)	1.0

* *p* value < 0.05, ** *p* value < 0.01 and *** *p* value < 0.001.

**Table 4:** Standard and comparison stimuli used in the discrimination experiment.

Standard (cm^2^/N)	Comparison (cm^2^/N)
0.125	0.059, 0.071, 0.091, 0.167, 0.2, 0.25, 0.33, 0.5, 1.0, 2.0
0.167	0.059, 0.071, 0.091, 0.125, 0.2, 0.25, 0.33, 0.5, 1.0, 2.0
0.2	0.059, 0.071, 0.091, 0.125, 0.167, 0.25, 0.33, 0.5, 1.0, 2.0
0.25	0.059, 0.071, 0.091, 0.125, 0.167, 0.2, 0.33, 0.5, 1.0, 2.0

## Data Availability

The original contributions presented in the study are included in the article, further inquiries can be directed to the corresponding author.
